# Zebrafish automatic monitoring system for conditioning and behavioral analysis

**DOI:** 10.1038/s41598-021-87502-6

**Published:** 2021-04-29

**Authors:** Marta de Oliveira Barreiros, Felipe Gomes Barbosa, Diego de Oliveira Dantas, Daniel de Matos Luna dos Santos, Sidarta Ribeiro, Giselle Cutrim de Oliveira Santos, Allan Kardec Barros

**Affiliations:** 1 Department of Electrical Engineering, Laboratory for Biological Information Processing (PIB), Federal University of Maranhão (UFMA), Av. dos Portugueses, 1966, Vila Bacanga, São Luís, MA CEP 65080-805 Brazil; 2 Brain Institute, Federal University of Rio Grande Do Norte, Av. Sen. Salgado Filho, 3000, Candelária, Natal, RN Brazil; 3 Department of Biology Sciences, State University of Maranhão, Cidade Universitaria Paulo VI S/N, São Luís, MA 65055-310 Brazil

**Keywords:** Computational science, Software, Engineering, Nanoscience and technology

## Abstract

Studies using zebrafish (*Danio rerio*) in neuro-behavioural research are growing. Measuring fish behavior by computational methods is one of the most efficient ways to avoid human bias in experimental analyses, extending them to various approaches. Sometimes, thorough analyses are difficult to do, as fish can behave unpredictably during an experimental strategy. However, the analyses can be implemented in an automated way, using an online strategy and video processing for a complete assessment of the zebrafish behavior, based on the detection and tracking of fish during an activity. Here, a fully automatic conditioning and detailed analysis of zebrafish behavior is presented. Microcontrolled components were used to control the delivery of visual and sound stimuli, in addition to the concise amounts of food after conditioned stimuli for adult zebrafish groups in a conventional tank. The images were captured and processed for automatic detection of the fish, and the training of the fish was done in two evaluation strategies: simple and complex. In simple conditioning, the zebrafish showed significant responses from the second attempt, learning that the conditioned stimulus was a predictor of food presentation in a specific space of the tank, where the food was dumped. When the fish were subjected to two stimuli for decision-making in the food reward, the zebrafish obtained better responses to red light stimuli in relation to vibration. The behavior change was clear in stimulated fish in relation to the control group, thus, the distances traveled and the speed were greater, while the polarization was lower in stimulated fish. This automated system allows for the conditioning and assessment of zebrafish behavior online, with greater stability in experiments, and in the analysis of the behavior of individual fish or fish schools, including learning and memory studies.

## Introduction

Learning and memory are complex brain processes that allow animals to adjust their behavior throughout the experience and in different environments^1,2^. In addition, learning and memory can help the fish improve anti-predator behavior when being attacked by predators by observing their companions' behavior^1,3^. Thus, learning plays an important role in the development of this behavior and has been studied in many animal models in the field of neuroscience and psychobiology^4–9^.

The zebrafish (*Danio rerio*) is a small freshwater teleost fish from South Asia^10^, and has become a model for behavioral research in neuroscience^5,11^, potentially suitable for studies on vertebrate learning^9^, because of the simplicity in the practice of maintenance, their proliferating nature and for having increased development of genetic markers, in addition to the practicality in handling, due to its small size (4 cm) and due to being fertile after three months. In addition to robust behavioral phenotypes, zebrafish has a homology with nucleotide and functional sequences among mammalian genes, which makes it suitable for research^12,13^.

Behavioral tests of learning and memory can be used even in the larval stage of zebrafish^14–16^. In this way, many experiences involving zebrafish behavior have already been tested and continue to be a field of study in research. In behavioral terms, several studies have shown zebrafish learning from different visual and sound approaches, such as identifying color preferences^13,17–19^, through the usage of lights (for association with danger, well-being, evacuation, memory^4,7^, in physical exercise^20^, in the usage of sounds to identify the fish's sound perception^21–24^, in the operant behavioral test to examine aspects of controlling a series of stimuli in zebrafish^6^, and several other approaches.

In addition, many behavioral studies have proposed measuring the level of fish learning by computational methods, based on fish detection and tracking, however, the biggest challenge of tracking algorithms is the complexity of fish movements during fast swimming captured in video images^1,4,12,25–31^. However, experiments that involve tracking fish positions in the video during the presentation of stimuli may allow a better analysis of the fully automated training of several adult zebrafish for arbitrary visual stimuli in parallel^18^. This way, automatic analysis of laboratory procedures maximizes data reliability, improving research performance and helping to standardize and validate experimental tests^5^.

Some test equipment has already been designed for fish conditioning, for example, a fully automated test system was designed to test aspects of impulse control in adult zebrafish, including hardware and software development^5,32,33^. In addition, an automated device enabled fast conditioning paradigms for zebrafish in five trials^7^. In another study, a robust self-administration trial of automated opioids for zebrafish, it was possible to measure the search for drugs, showing a characteristic of drug dependence and information on the underlying biological pathways^34^.

The quantification of behavioral responses can assess different situations in conjunction with stimuli or stressors. Thus, it can be considered a good indicator of the body’s responses^35,36^. Therefore, it is important that the fish conditioning equipment is fully automatic and has a design adjusted to the procedure. A fully automatic procedure can reduce the human bias during the analysis of the research, as many procedures require maintenance of the environment at certain times, such as the control of food, oxygenation or stimuli activated in the tank (lights or sounds). In this, the automation of procedures and analysis of videos to explore the experimental extension is very favorable for research in the behavioral area^5^. By obtaining a standardized training protocol, comparison of results in different experiments and laboratories is facilitated, giving better usability of zebrafish in behavioral research^18^.

Although there are several conditioning/training methods and some computational algorithms available for zebrafish behavioral assessment, there are still few complete systems for conditioning and automatic assessment of fish behavior. Typically, these behavioral assessment software are developed separately from the automatic conditioning hardware, limiting themselves to capturing videos at the time of training, for later analysis. Here, we describe the technical development of fully controlled hardware and an automated algorithm that allows for the control of protocols for conditioning and assessing the behavior of the fish, which involves the delivery of food and stimuli, being a tool developed for the analysis of video processing of adult fish in real time. In addition, this tool allows for changing the settings for new tests and can be used for other applications involving the behavior of fish, as well as assessing the behavior for toxicity, sleep, aversion and several others.

## Materials and methods

### Animals

Forty-three adult zebra fish (mixed sex, size 3 cm ± 0.5 cm) were obtained from a local pet store (São Luís, State of Maranhão, Brazil), being housed in three groups, a group with 11 fish for simple learning (single stimulus), a group with 16 fish for complex learning (two stimuli: vibracall and light) and a control group with 16 fish in total, used for both experiments. The groups of fish were housed in a 22.5L (250 × 200 × 550m m) opaque glass tank with aerated and filtered water. The fish were housed in a closed system with filtered water (sponge and activated carbon), being given two weeks to acclimatize in the environment before the established training. The water quality was monitored weekly, keeping the temperature at 26 ± 1 °C, pH 7.2 ± 0.3, ammonia 0.00 ± 0.01 ppm, nitrite 0.00 ± 0.01, nitrate ~ 40 ppm and conductivity ~ 1250 µS, with mechanical, biological and chemical filtration. The water was changed (40%) every 10 days to guarantee the quality. The lighting was adjusted in a 12/12 h light/dark cycle^23,37,38^. The fish were fed four times a day with commercial foods and crushed flakes (38% protein, 4% lipids, Nutricom Pet). In the period of maintenance or acclimation of the fish in the tank, the fish were fed twice a day, ad libitum, but during conditioned training feeding was restricted (~ 2 g each training).

### Ethics statement

All experimental procedures with animals followed the ARRIVE^39^ guidelines and approved by the Ethics Committee on Animal Use (CEUA) of the Federal University of Maranhão (UFMA), campus of São Luís—MA, Brazil (Protocol nº 23115.006194/2018-01 and Protocol n° 23115.024712/2020-96). All experiments were performed in accordance with relevant named guidelines and regulations. All training was carried out in the same environment where the fish were kept. After the entire experiment, the euthanasia method was used, utilizing an anesthetic solution of benzocaine type 60–70 mg/L of water with subsequent cooling and freezing of the carcass at – 20 °C, discarded according to the protocol of the Bioterium of the University.

### Experimental setup

The behavioral system consisted of hardware and software to manage the experimental workflow and video processing. The experimental strategy consisted of carrying out associative learning tasks, with visual and sound stimuli associated with food, in addition to assessing the response of the zebrafish in decision making during conditioning activities. The behavioral modeling of zebrafish groups and the dynamics of fish behavior in different groups were analyzed in this configuration.

### Hardware

An experimental rack was designed in a room, having three opaque glass tanks 22.5L of 550 mm × 200 mm × 250 mm (width × height × depth), containing three groups of fish, connected by a computer for automatic control of the training, acclimation, oxygenation and feeding of the fish. The tank was divided into two arenas: oxygenation space (100 mm × 250 mm × 200 mm) and training space (450 mm × 200 mm × 250 mm). 8 mm holes were created in the internal wall of the tank that separated the two environments, so that the oxygenated water circulated in the fish training environment without bubbles or interference in the video analysis.

The automatic control of the tank was done by microcontrolled components (using Arduino MEGA), controlling the oxygenation of the fish, the ambient light, the light stimulus and the feeder, obeying the schedule of training activities (four times a day). Automatic feeders were produced on the 3D printer, using Acrylonitrile Butadiene Styrene (ABS) filaments, being coupled to the outside and top of the tank. The food was placed in the feeder reservoir and could be dispensed using a servomotor, which turned a gear at the feeder outlet to have a uniform control of the amount of ground feed (about ~ 2 g).

**Figure 1:**
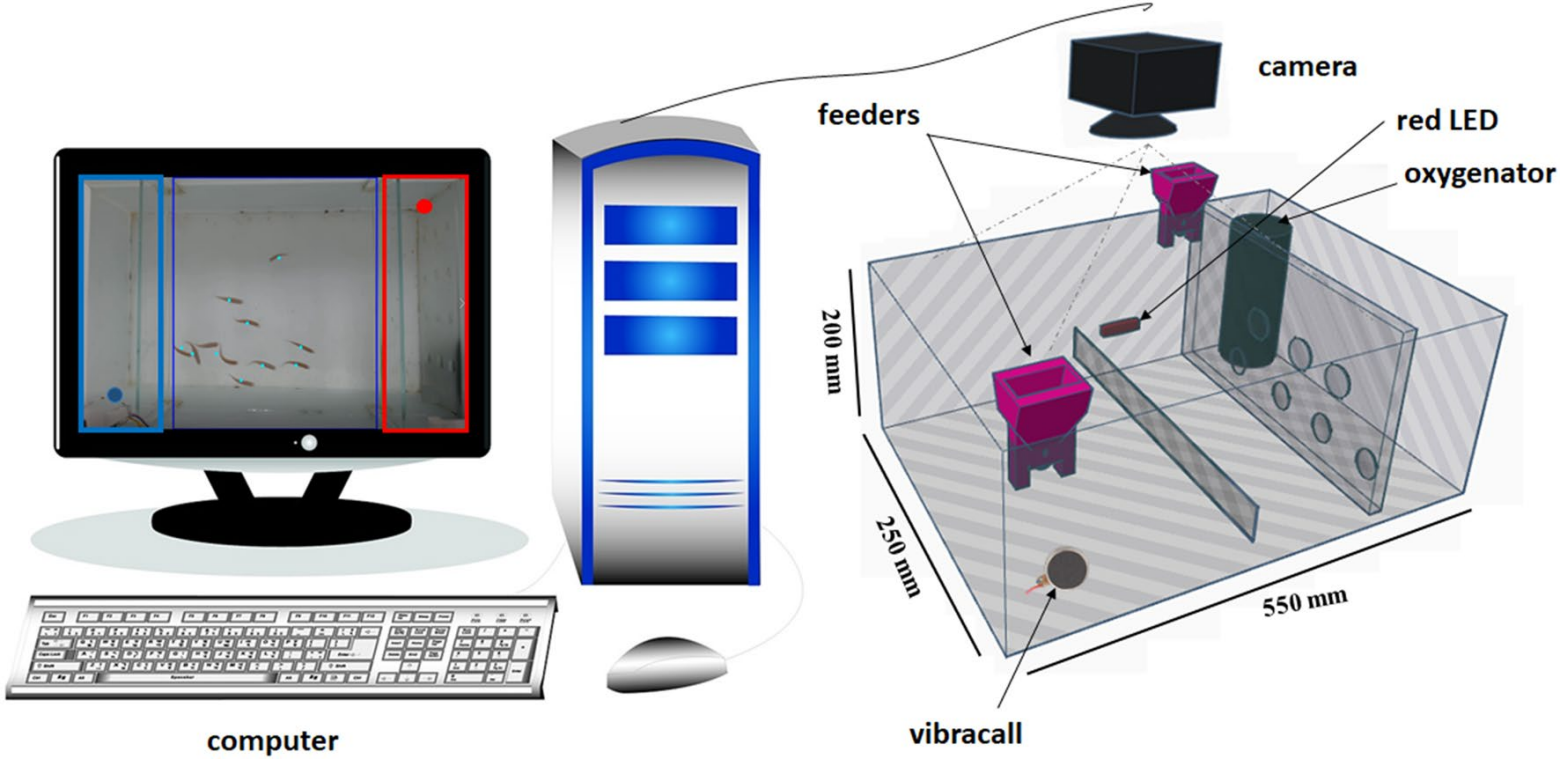
Experimental configuration to measure zebrafish learning. The group of zebrafish swims in a sandblasted glass tank, divided into two feeding arenas. A red LED strip is attached to the external area on the right side of the tank as a light stimulus. And a vibracall was attached to the external area on the left side of the tank. A camera is mounted above the tank, approximately 40 cm.

The internal division in the tank was adjusted according to the activity established at that moment and with the proposed experiments. Thus, the glass partitions (250 mm × 80 mm × 4 mm) were added inside the tank, in order to separate the space from the food, during food conditioning. In addition, a red LED strip was connected during the associative activity for the simple experiment. Two stimuli were adjusted for the complex experiment, red color (spectrum ~ 663 nm) and vibracall, placed in the external area in the aquarium. Red lighting was chosen based on the sensitivity of the zebrafish, where zebrafish favor red over other colors. However between the green and blue colors, green was the most preferred, with the fish having a strong aversion to the blue color^13,17,19,40^. For simple conditioning, only one divider was used to separate the food from the remaining space in the tank. And a visual stimulus in red color was offered to the fish during the conditioning phase in the feeding period. Oxygenation was kept on in the tank, but turned off 10 min before the beginning of the tests so that there was no flow of bubbles in the water, being turned on again 10 min after the tests, following a schedule of activities for training, implemented automatically. In experiments that did not consist of food conditioning, the tank had no division and the fish could swim normally in the entire tank. And a single camera was mounted on each tank approximately 40 cm above each tank to capture the videos of the proposed training. The videos were recorded at 30 frames per second with a resolution of 1920X1080 pixels (C920 Logitec camera). The focus of the camera was set manually, adjusting the dimensions of the tank (Fig. 1).

For the control of the hardware, scripts were produced in Matlab and Python for the total control of the automatic conditioning system. This mechanism served to deliver food, turn on and off ambient light, control the oxygen pump and activate stimuli at the set time of the experiments.

### Software

The behavioral evaluation algorithm consisted of the following stages: image processing for fish detection and tracking, behavioral modeling and automatic analysis of fish conditioning in the interest of the stimulus (see supplementary file S1).

#### Detection and tracking

In the fish processing and detection phase, the videos were processed by an algorithm based on YOLOv2 convolutional network^41–43^. The architecture of the YOLOv2 network consisted of seven convolution layers with a 3 × 3 filter, six layers of Relu, three layers of Max Pooling with size 2 × 2, six layers of batch normalization with size 1 × 1 and two layers of anchor boxes. Table 1 shows the architecture of the YOLOv2 network used to detect fish in the tank. For the training of the YOLOv2 network, a set of images was used containing 365 images referring to previous videos of the fish, where the fish head region was manually marked on all images, being used for learning of the YOLOv2 network for the detection of fish in the tank. Then, the trained net was used to detect the fish during the behavioral experiment. For more details, see the reference by Barreiros et al.^43^.

**Table 1:** YOLOv2 network architecture parameters for zebrafish detection.

Layer	Operation	Filters	Kernel size	Stride	Activations
1	Convolution—ReLu	16	3 × 3	1	480 × 480 × 16
	Batch normalization	16	1 × 1	–	480 × 480 × 16
2	Max pooling	–	–	2 × 2	240 × 240 × 32
	Convolution—ReLu	32	3 × 3	1	240 × 240 × 32
	Batch normalization	32	1 × 1	–	240 × 240 × 32
3	Max pooling	–	–	2 × 2	120 × 120 × 64
	Convolution—ReLu	64	3 × 3	1	120 × 120 × 64
	Batch normalization	64	1 × 1	–	120 × 120 × 64
4	Max pooling	–	–	2 × 2	60 × 60 × 128
	Convolution—ReLu	128	3 × 3	1	60 × 60 × 128
	Batch normalization	128	1 × 1	–	60 × 60 × 128
5	Convolution—ReLu	128	3 × 3	1	60 × 60 × 128
	Batch normalization	128	1 × 1	–	60 × 60 × 128
6	Convolution—ReLu	128	3 × 3	1	60 × 60 × 128
	Batch normalization	128	1 × 1	–	60 × 60 × 128
7	Convolution—ReLu	24	1 × 1	1	60 × 60 × 24
	Anchor	24	–	–	60 × 60 × 24

To adjust the detection accuracy of the YOLOv2 network and establish the tracking of fish during activities in the tank, a simple Kalman filter was previously implemented^43^, and configured to estimate the coordinates of the fish in the detection failure, during the times of occlusions by other fish during swimming, thus, the loss of detection could be recovered. Then, after estimating the position of the fish, a cost function was adjusted and minimized, associating the estimated detection with the actual detection of the YOLOv2 network. In case of loss by the detector or occlusion, the Kalman filter adjusts the new estimated position, detecting each fish in the consecutive frames to be linked when finding the shortest distance and the angle of the fish's head direction in the detection of the YOLOv2 and in the estimation of the Kalman filter, thus associating it with the corresponding fish and allowing the creation of paths for each fish in the frame over time. The identification of the fish is initially chosen automatically, but does not guarantee that the following videos will detect the same fish. Thus, from these steps, it was possible to measure the behavioral levels of the fish individually and separated into groups during the behavioral testing activities, such as polarization, group dynamics, distance covered, speed and tracking of the route of the shoal.

#### Behavioral modeling of zebrafish

To assess the complex behavior of the fish in group and individually, we have implemented some evaluation metrics, as described below.

##### Polarization

 To measure the degree of coordination of the group of fish in each frame, the polarization pol was used, which quantifies the intensity of the parallel orientation in a school of fish, defined by Eqs. (1), (2) and (3):

1 $$pol\left( t \right) = \left| {M\left( t \right)} \right|$$

2 $$M\left( t \right) = \frac{1}{N}\mathop \sum \limits_{k = 1}^{N} \hat{v}_{k} \left( t \right)$$

3 $$\hat{v}_{k} \left( t \right) = \frac{{v_{k} \left( t \right)}}{{\left| {v_{k} \left( t \right)} \right|}}$$

where pol(t), is the arithmetic mean of the unit direction of all fish in frame t; $\hat{v}_{k} \left( t \right)$ is the unit direction of fish k in frame t; $v_{k} \left( t \right)$ is the velocity of the fish k in frame t; N the total number of fish in the group.

##### Speed

 To evaluate the locomotion of the fish in the tank and the processing speed of the acquired information, the scalar speed was used, calculated from the relation of its displacement in relation to time, given by Eq. (4).

4 $$vel_{k} \left( t \right) = \frac{{\sqrt {\left( {x_{k,t} - x_{k,t - 1} } \right)^{2} + \left( {y_{k,t} - y_{k,t - 1} } \right)^{2} } }}{\Delta t}$$

where $vel_{k} \left( t \right)$ is speed of the k-th head of the fish in frame $t$, $x_{k,t}$ and $y_{k,t}$ the *x* and *y* position k-th fish head in frame $t$, $x_{k,t - 1}$ and $y_{k,t - 1}$ the *x* and *y* position k-th fish head in frame $t - 1$ and $\Delta t$ is the time in seconds between frame capture.

##### Fish interaction network

 Collective behavior is the result of different dynamics and is sometimes compared to living systems. When a group of fish move, these animals usually process information to coordinate their movements. Individuals respond dynamically and can make good decisions as a collective to changing environments^44^. Recent studies in information theory have started to apply methods to quantify this distributed processing^45–47^. Here, we represent the school's interaction network as a directed graph, using mutual information data to quantify this distributed processing. In order to calculate the influence of a fish's movement in relation to the other fish in the tank, the variation of the direction of the fish head for the duration of the established time is utilized. It is expected that the fish with the highest rate of interaction with the other fish in the tank can influence the dynamics of the school. To measure the information between a fish and its neighbor, Mutual Information is used, as described in Niizato et al.^46^. The values of mutual information $MI\left( {.,.} \right)$ were calculated in pairs with all combinations of fish, and the origin and destination fish ratios are stored in a matrix by Eq. (5).

5 $$Mutualinfo = \left[ {\begin{array}{*{20}c} 0 & {MI\left( {{\Phi }_{1} ,{\Phi }_{2} } \right)} & \ldots & {MI\left( {{\Phi }_{1} ,{\Phi }_{N} } \right)} \\ {MI\left( {{\Phi }_{2} ,{\Phi }_{1} } \right)} & 0 & \ldots & {MI\left( {{\Phi }_{2} ,{\Phi }_{N} } \right)} \\ \vdots & \vdots & \vdots & \vdots \\ {MI\left( {{\Phi }_{N} ,{\Phi }_{1} } \right)} & {MI\left( {{\Phi }_{N} ,{\Phi }_{2} } \right)} & \ldots & 0 \\ \end{array} } \right]$$

where $MutualInfo$ is the matrix that stores the relationship of mutual information in relation to all pairs of fish, the value of all diagonal elements is equal to zero and $MI\left( {.,.} \right)$ is the mutual information function. Where ${\Phi }_{k}$ is the time series of the variation in the direction of the head of the k-th fish and N is the total number of fish. Mutual information is symmetrical, that is, the value of $MI\left( {{\Phi }_{1} ,{\Phi }_{2} } \right)$ is equal to $MI\left( {{\Phi }_{2} ,{\Phi }_{1} } \right)$.

#### Automatic conditioning analysis

To analyze the conditioning of the fish in the videos, the image of the tank was divided into arenas for simple conditioning (feeder and swimming area) and four arenas of equal size for complex training in the shape of a cross, where each arena corresponded to a 22.5 cm × 12.5 cm rectangle (Fig. 3F), one area corresponds to the red light stimulus and another to the vibracall stimulus. Then, fish detection was automatically established during conditioning in the tank's region of interest. Two types of videos were automatically saved in a folder, one video with the detection mark and the other without any type of detection, for later analysis. In addition, a heat map was created containing the locations of the fish on the frames over time. The heat map is a matrix, where each cell represents a pixel batch of the area to be analyzed. The colors of the heat map indicate the intensity that a given event occurred in the batch. In this case, the aquarium (45 cm × 25 cm, width × depth) was used as a 10 × 10 matrix, dividing its surface into pixel lots, equivalent to 4.5 cm and 2.5 cm each lot. Each portion is represented by a cell in the heat map matrix and the intensity of the cell is defined by the number of fish head detections in a batch, from a set of frames in the video. After counting occurrences of fish detection in the flocks, the heat map is subjected to a low-pass filter (Gaussian filter) to soften the map image.

### Experiment 1: evaluation of simple conditioning with a single stimulus

For simple conditioning, fish (n = 11) had to associate a single light stimulus to the feeding period. The tank was adjusted to contain a divider that separated the food from the remaining space of the tank. Then, a red LED strip (spectrum ~ 663 nm) was turned on 30 s before the food was poured from the feeder, the red LED was located on the same side of the feeder in the tank. The conditioning of the zebrafish consisted of four daily sessions with an interval of 3 h during the biological experiment (the time was defined in order to obtain the same interval between all attempts). All experiments were established at the same time (8:00 am, 11:00 am, 2:00 pm and 5:00 pm) every day, to avoid any daytime effects, configured to the automatic conditioning system. The training took place at the time of feeding to reinforce the association with the stimulus. The result of laboratory procedures on the behavior of zebrafish was tested against the control group (undisturbed fish). The behavioral assessment of the fish consisted of intervals with two minutes of video recording at each stimulus, divided into four consecutive periods: 30 s before the light stimulus, 30 s during the light stimulus, 30 s during feeding and 30 s after the light stimulus was turned off. Simple training was established in just one day, containing four attempts at appetite conditioning.

### Experiment 2: evaluation of complex conditioning with two stimuli

For complex conditioning, a new group of fish (n = 16) was subjected to conditioning containing two stimuli in the feeding period: light and vibracall. The tank was adjusted to contain a glass partition placed at the bottom of the aquarium to separate the two sides of the zebrafish food conditioning and to make access to the feeder more difficult. In this experiment, a vibracall and a red LED light were used. The red LED was located on the bottom of the aquarium, located on the right side, whereas the vibracall was located on the left side of the aquarium, positioned on the outside and at the bottom of the aquarium (see Fig. 1). Each stimulus corresponded to one side of the dump of food in the feeder, and the fish would need to know which color corresponded to the correct side of the feed at that time. Thus, two automatic feeders were adjusted to synchronize the feed with the light stimulus. The behavioral assessment of the fish in this training was analyzed for one minute of video recording at each stimulus, divided into four observatory periods: 20 s before the light stimulus, 15 s during the light stimulus, 15 s during feeding and 20 s after the stimulus was turned off. The complex training was established by 10 attempts for each stimulus, in alternate periods so that the fish did not learn the sequence of the stimuli. Fish that managed to stay close to the enclosure in a timely manner, were responding to stimuli at that time. Thus, the zebrafish was considered to be successfully conditioned when it was directed to the food arena during the light phase that preceded the feeding. And, all experiments were established at the same time (8 am, 11 am, 2 pm and 5 pm) automatically by the conditioning system, in order to avoid any daytime effects. The fish considered conditioned were only the fish that were in an area with a dimension of 12.5 cm × 22.5 in the corner corresponding to the stimulus (see Fig. 3F).

### Experiment 3: behavioral modeling in conditioned fish

During complex conditioning (containing two stimuli), the polarization of the fish group during the established attempts was calculated to observe the change in the behavior of the group when they were stimulated in relation to the control group (not stimulated). In addition to the measurement of polarization, the fish were tracked, measuring the speed and the average distance of the fish traveled in the tank, thus, it was observed the level of change in the behavior of the fish and their interest in relation to the stimulus. The analyses were made only when the stimulus was lit, prior to feeding. In addition, for comparative purposes, we group the fish in different sizes 3, 6, 8, 11 and 16 to verify the behavior by different groups, at the moment the light was turned off.

### Experiment 4: behavioral dynamics of zebrafish groups

To measure the influence of a fish's movement in relation to the other fish in the tank, a video set was used in which the fish were grouped in different sizes (3, 5, 8, 11 and 16 (see supplementary information S2)), freestyle, referring to experiment 2. In addition, videos referring to the conditioning of fish with two stimuli were used in all attempts. Thus, it was verified how the fish could influence the dynamics of the group or if only one fish could coordinate the same group during free swimming.

### Control

For the control group, a third new group was formed by 16 zebrafish, the light and vibracall stimuli were offered to the tank in random periods, different from the feeding period, observing the behavior of the fish during the lit light stimulus. All procedures were performed in the same setting tank as the fish groups.

### Statistical analysis

The conditioned response of the fish to the consecutive stimuli on the experimental day was compared. Statistical analyzes of the data were performed using SPSS software (Statistical Package for the Social Sciences, Inc., Chicago, IL, USA) version 25.0. Graphs were performed using PRISM8 (Graph-Pad) software. The Kolmogorov–Smirnov test was used to verify the normality of the data. The ones used for non-parametric statistics were: Mann–Whitney U-tests (MW), to compare groups of fish conditioned with a single stimulus; Friedman (H) tests were used to assess the progression of each group. The parametric statistics were used to compare the groups of fish stimulated with two stimuli (red light and vibracall), using the Student t test (Test t) to compare the number of fish located in the area defined for conditioning in the tank, in addition to speed, distance and polarization of groups of fish conditioned with two stimuli. One-Way ANOVA (F) was used to assess the progression of each group in all periods. For all tests, the alpha level was adjusted to 0.05. The response capacity of the zebrafish was measured as the average increase in the number of zebrafish in the food arena after the initiation of the light stimulus.

## Results

### Simple conditioning

**Figure 2:**
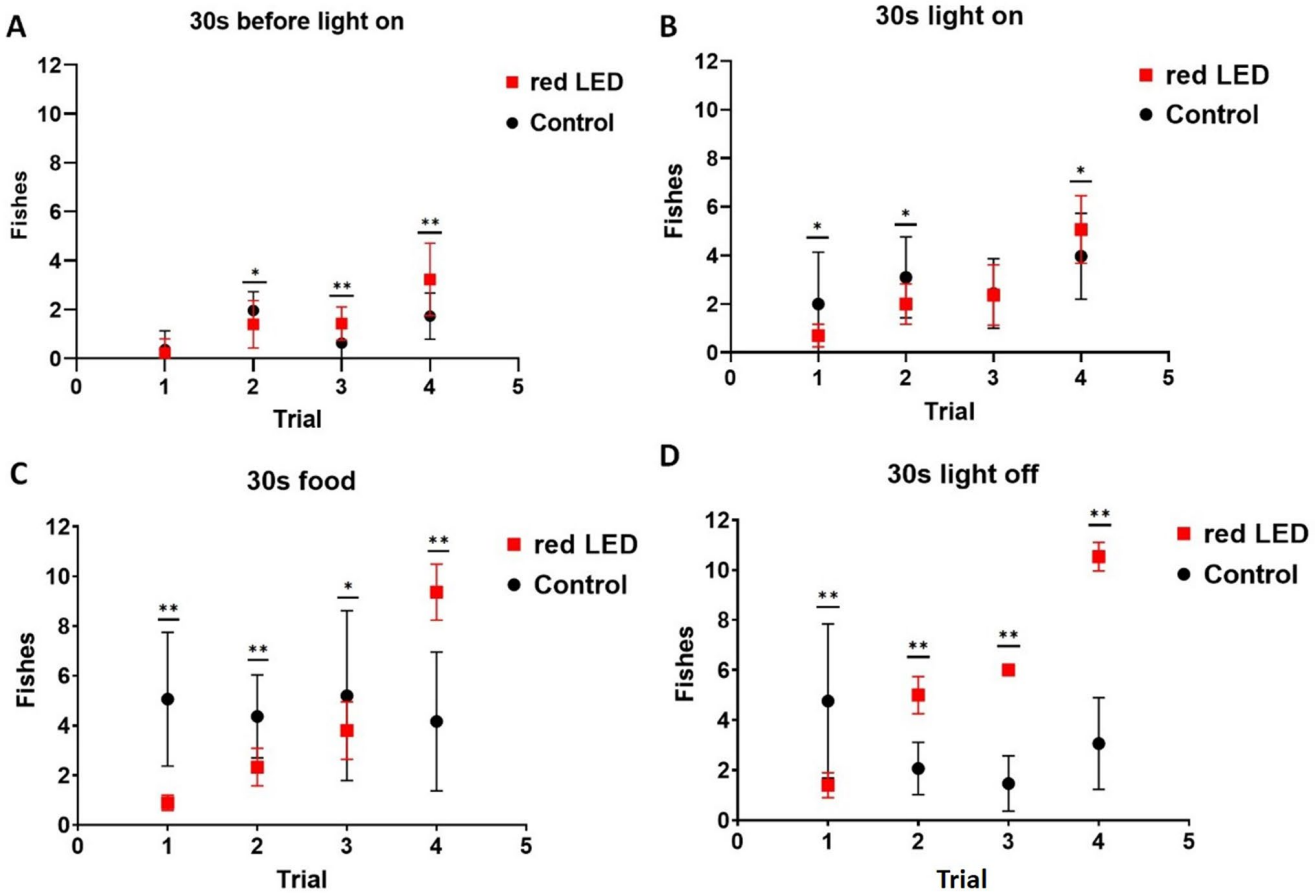
Zebrafish simple appetite conditioning responses using one stimulus: Red LED light. (**A**) Number of fish that were in the eating behavior before the light was turned on, (**B**) while the light was on, (**C**) during the feeding period and (**D**) after the stimulus is turned off. The horizontal lines of each group denote the mean ± SD and the asterisks on the brackets indicate statistical difference in comparison to the frequency of fish between the attempt and the control group by Mann–Whitney U-test (*p < 0.05; * *p < 0.001, ***p < 0.0001).

In the first experiment, 11 adult zebrafish were used, conditioned with a single red stimulus during the feeding period. The experimental results of this study are shown in Fig. 2. In the first attempt, during the period of the LED light off (Fig. 2A), there was no association between the group of trained fish and the control group (MW: U = 418.5; *p* = 0.504). However, the position of the fish before the light stimulus differed in the other attempts in the control group (Friedman: H = 50.61; *p* < 0.001) and in the stimulated group (Friedman: H = 60.18; *p* < 0.001). The fish progressed a lot in other attempts of associative learning with a single stimulus (red LED) (Fig. 2B), managing to quickly associate the conditioned stimulus from the second attempt (30 s during the connected stimulus), between the stimulated group and the control group (trial 1: MW: U = 307.50; *p* = 0.025; trial 2: MW: U = 258.0; *p* = 0.003; trial 3: MW: U = 437.0; *p* = 0.843; trial 4: MW: U = 270.0; *p* = 0.007). The number of fish increased after the light signal during the trials; both in the stimulated group and in the control group (Friedman in the stimulated group: H = 71.76; *p* < 0.001; Friedman in the control group: H = 18.78; *p* < 0.001).

In addition, initially, during the feeding period (Fig. 2C), most fish were unable to access the food compartment in a timely manner, however, as each attempt was made, the fish learned the route to access the food arena, passing under the divider demarcated at the precise time. There was a significant difference in the progression of conditioning attempts between the stimulated group and the control group (trial 1: MW: U = 69.00; *p* < 0.001; trial 2: MW: U = 117.5; *p* < 0.001; trial 3: MW: U = 298.50; *p* < 0.024; trial 4: MW: U = 65.0; *p* < 0.001). And there was a change in the behavior of the fish during the attempts, both in the stimulated group and in the control group (Friedman of the stimulated group: H = 88.72; *p* < 0.001; Friedman of the control group: H = 10.728; *p* < 0.013).

After the training ended, at each attempt, the fish remained in the reserved space waiting for more food, suggesting that the fish understood that there was a space reserved for food, and expected more food to be released after the light was turned off (Fig. 2D). Therefore, there was a significant difference between the stimulated group and the control group (trial 1: MW: U = 204,00; *p* < 0,001; trial 2: MW: U = 8,0; *p* < 0,001; trial 3: MW: U = 0,00; *p* < 0,001; trial 4: MW: U = 0,00; *p* < 0,001). In addition, there was a change in fish behavior during attempts for the stimulated group and the control group (Friedman in the stimulated group: H = 88.19; *p* < 0.001; Friedman in the control group: H = 20.96; *p* < 0.001).

### Complex conditioning: red light and vibracall

In the second experiment, 16 new zebrafish were used for the training considered complex, where the fish should recognize two stimuli: LED light (red color) and vibration, in order to make a correct decision to go to the side of the corresponding tank (right or left side) to the triggered stimulus. There were 20 total attempts, alternating stimuli (red LED and vibracall) during training.

**Figure 3:**
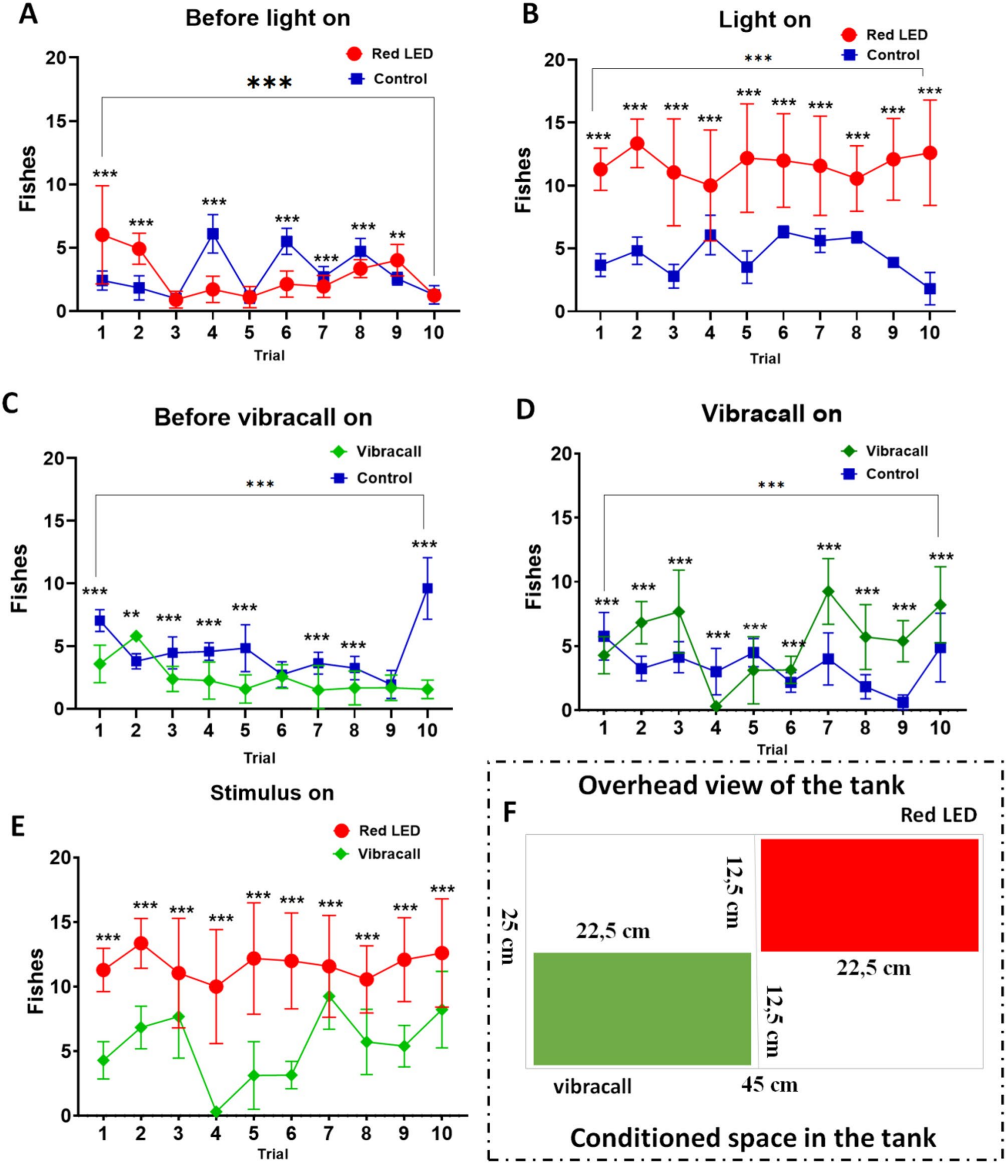
Responses of zebrafish complex appetitive conditioning using two stimuli: red LED light and vibration. (**A**) Number of fish that were in the feeding behavior before the light was turned on. (**B**) Number of fish conditioned with red LED light stimulus during the attempts. (**C**) Number of fish in the food compartment before the vibracall stimulus is activated. (**D**) Number of fish conditioned with vibracall stimulus during the attempts. (**E**) Comparison of the number of fish conditioned by light and vibracall. (**F**) Top view of the tank referring to the conditioning area (red area: red LED and green area: vibracall). The horizontal lines of each group denote the mean ± SD and the asterisks on the brackets indicate statistical difference in comparison with the frequency of fish between the attempt and the control group by student t test (*p < 0.05; **p < 0.001, ***p < 0.0001).

Before the red LED lights up (Fig. 3A), the behavior of the fish was observed for comparative purposes. There was a progression of behavior in a non-linear way between the initial attempt (trial 1: 6.5 ± 3.8 (mean ± SD), variation of 58.56%) and the last attempt (trial 10: 1.58 ± 0, 43 (mean ± SD), variation of 34.91%), t test = 14.696, df = 149, *p* < 0.0001, but there was a change in the behavior of the fish between all attempts (${F}_{\mathrm{9,13}}$ = 218.0, *p* < 0.0001). However, during the period in which the red LED was on, the zebrafish showed a strong change in behavior between the stimulated group and the control group in all attempts (trial 1: test t = − 43.77, *p* < 0.0001; trial 2: test = − 51.51, *p* < 0.0001; trial 3: − 29.57, *p* < 0.0001; trial 4: test t = − 13.09, *p* < 0.0001; trial 5: − 28.25, *p* < 0.0001; trial 6: test t = − 17.08 *p* < 0.0001; trial 7: test t − 19.79, *p* < 0.0001; trial 8: test t = − 28.45, *p* < 0.0001; trial 9: test t = − 33.31, *p* < 0.0001; trial 10: test t = − 33.71, *p* < 0.0001), the fish associated the stimulus to the feeding period quickly, shifting to the space reserved for feeding (Fig. 3F, red area), showing that the behavior of the fish has changed in all attempts, having a significant progression during training (${F}_{\mathrm{9,13}}$ = 11.55, *p* < 0.0001) (Fig. 3B).

The results of the tests with the vibration are shown in Fig. 3C and D. Before the vibration was triggered (Fig. 3C), the fish were only a few times in the food compartment (Fig. 3F, green area), with a non-linear behavior between the initial attempt (trial 1: 4.29 ± 1.44 (mean ± SD), variation of 33.55%) and the last attempt (trial 10: 8.22 ± 2.96 (mean ± SD), variation of 36.01%), t test = 15.424, df = 149, *p* < 0.0001, but there was a change in the behavior of the fish between all attempts (${F}_{\mathrm{9,13}}$ = 180.0, *p* < 0.0001). In the period stimulated with activated vibration (Fig. 3D), the fish were able to associate the vibracall stimulus with the feeding period, with a significant change in the behavior of the stimulated fish in relation to the control group in all attempts (trial 1: test t = 7.69, *p* < 0.0001; trial 2: test = − 23.50, *p* < 0.0001; trial 3: − 10.97, *p* < 0.0001; trial 4: test t = 16.03, *p* < 0.0001; trial 5: 7.43, *p* < 0.0001; trial 6: test t = − 9.85 *p* < 0.0001; trial 7: test t − 23.38, *p* < 0.0001 ; trial 8: test t = − 14.13, *p* < 0.0001; trial 9: test t = -37.08, *p* < 0.0001; trial 10: test t = − 10.20, *p* < 0, 0001). In all training periods, during the triggered stimulus, there was a significant progression in the behavior of the fish (${F}_{\mathrm{9,13}}$ = 236.5, *p* < 0.0001).

It is observed that the zebrafish learned to associate the red light stimulus more quickly than the vibration stimulus (Fig. 3E). There was a strong significant difference in all trials (trial 1: test t = 75.92, *p* < 0.0001; trial 2: test = 28.89, *p* < 0.0001; trial 3: 10.01, *p* < 0.0001; trial 4: test t = 28.39, *p* < 0.0001; trial 5: 25.05, *p* < 0.0001; trial 6: test t = 33.28 *p* < 0.0001; trial 7: test t = 7.36, *p* < 0.0001; trial 8: test t = 27.79, *p* < 0.0001; trial 9: test t = 29.87, *p* < 0.0001; trial 10: test t = 21.21, *p* < 0.0001). In addition, the behavior of the fish appeared to be more stable in all attempts while the light was on (mean ± SD: 11.67 ± 0.98, variation of 8.45%) than stimulation by vibracall (mean ± SD: 5.39 ± 2.74, 50.76% change).

### Change in the behavior of fish conditioned with two stimuli

**Figure 4:**
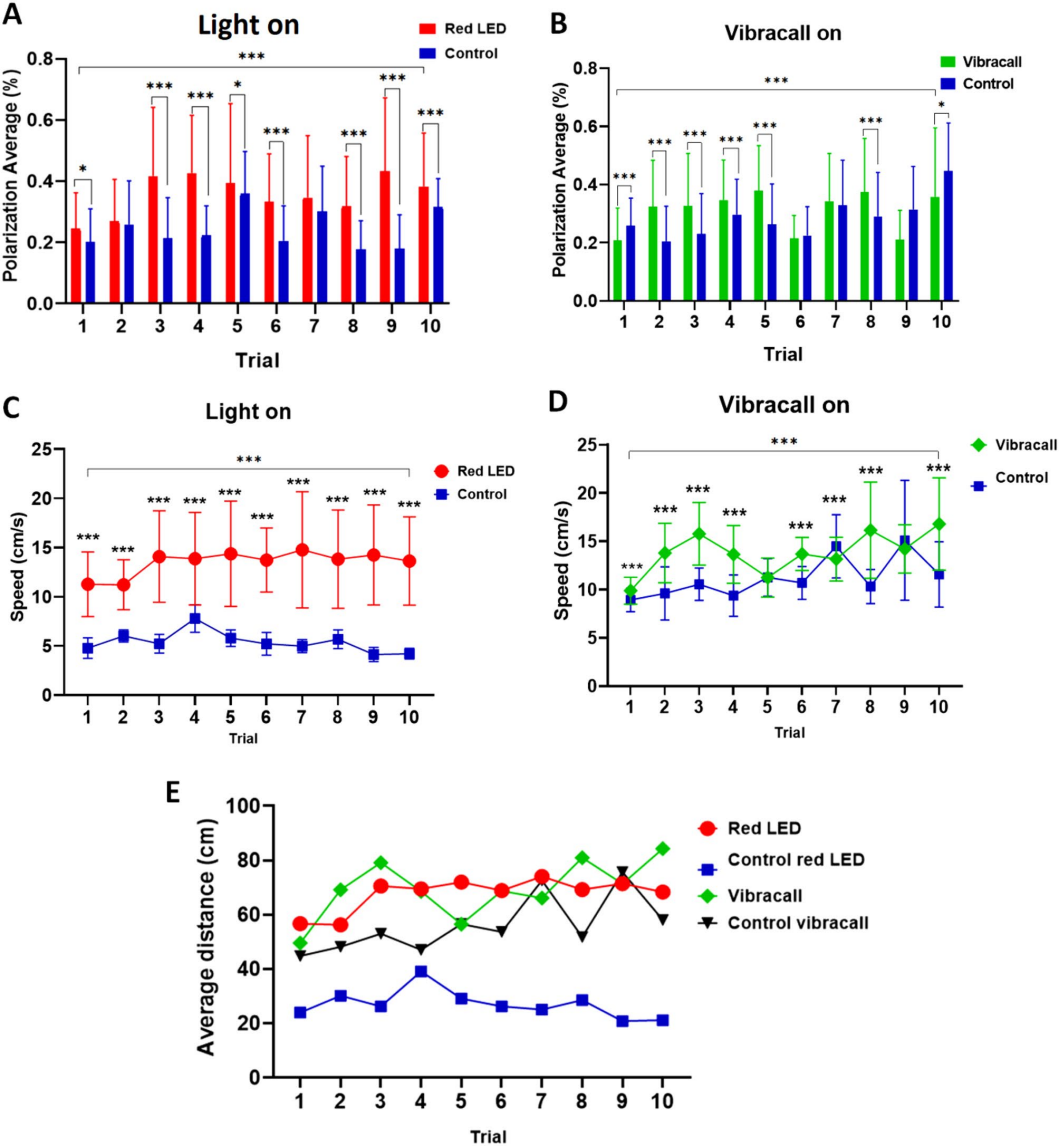
Zebrafish group behavior responses stimulated with red LED light and vibration. (**A**) Average polarization of the zebrafish groups during attempts at conditioning with red light and control group. (**B**) Average polarization of the zebrafish groups during attempts at conditioning with vibracall and control group. (**C**) Average speed covered by the zebrafish groups during conditioning with red light and control group. (**D**) Average speed traveled by the zebrafish groups during conditioning with vibracall and control group. (**E**) Relation of the distance traveled between the groups of fish stimulated with light, vibracall and control. The horizontal lines of each group denote the mean ± SD and the asterisks on the brackets indicate statistical difference in comparison with the frequency of fish between the attempt and the control group by student t test (*p < 0.05; **p < 0.001, ***p < 0.0001).

The degree of coordination of the zebrafish group was measured to quantify the intensity of the parallel orientation of the fish school. In all attempts at conditioning using the red light stimulus, we found a greater polarity between the zebrafish groups stimulated in relation to the control group (trial 1: test t = 3.21, *p* = 0.002; trial 2: test = 1.077, *p* = 0.283; trial 3: 11.76, *p* < 0.0001; trial 4: test t = 10.47, *p* < 0.0001; trial 5: t = 2.10, *p* = 0.037; trial 6: test t = 7.59 *p* < 0.0001; trial 7: test t = 1.92, p = 0.057; trial 8: test t = 7.81, *p* < 0.0001; trial 9: test t = 10.50, *p* < 0.0001; trial 10: test t = 4.41, *p* < 0.0001) (Fig. 4A). However, the polarization of the fish during the connected vibracall seemed to be more variable than in the red stimulus. In some trial attempts there were no significant differences between the stimulated group and the control group (trial 6: test t = − 1.13, *p* > 0.05; trial 7: test t = 0.757, *p* > 0.05) (Fig. 4B).

Fish stimulated with the red LED stimulus were faster in all conditioning attempts compared to the unstimulated group (trial 1: test t = 24.01, *p* < 0.0001; trial 2: test = 25.46, *p* < 0.0001; trial 3: 23.64, *p* < 0.0001; trial 4: test t = 16.022, *p* < 0.0001; trial 5: t = 18.39, *p* < 0.0001; trial 6: test t = 19.79 *p* < 0.0001; trial 7: test t = 19.60, *p* < 0.0001; trial 8: test t = 24.52, *p* < 0.0001; trial 9: test t = 25.39, *p* < 0.0001; trial 10: test t = − 5.26, *p* < 0.0001), the zebrafish were able to access the area marked for fast-progress feeding, showing that the fish understood that it was the period prior to feeding (${F}_{\mathrm{9,13}}$ = 11.11, *p* < 0.0001) (Fig. 4C). However, the fish stimulated by vibracall showed some instabilities in the speed covered in relation to the control group, and in some attempts they did not present significant differences (trial 5: test t = − 0.028, *p* = 0.978; trial 9: − 1.87, *p* = 0.063), but there was a change in behavior during all attempts (F (9, 1490) = 70.03, *p* < 0.0001) (Fig. 4D). In addition, stimulated fish traveled more through the tank during stimulated conditioning attempts compared to fish in the control group (${F}_{\mathrm{3,36}}$ = 53.48, *p* < 0.0001) (Fig. 4E).

**Figure 5:**
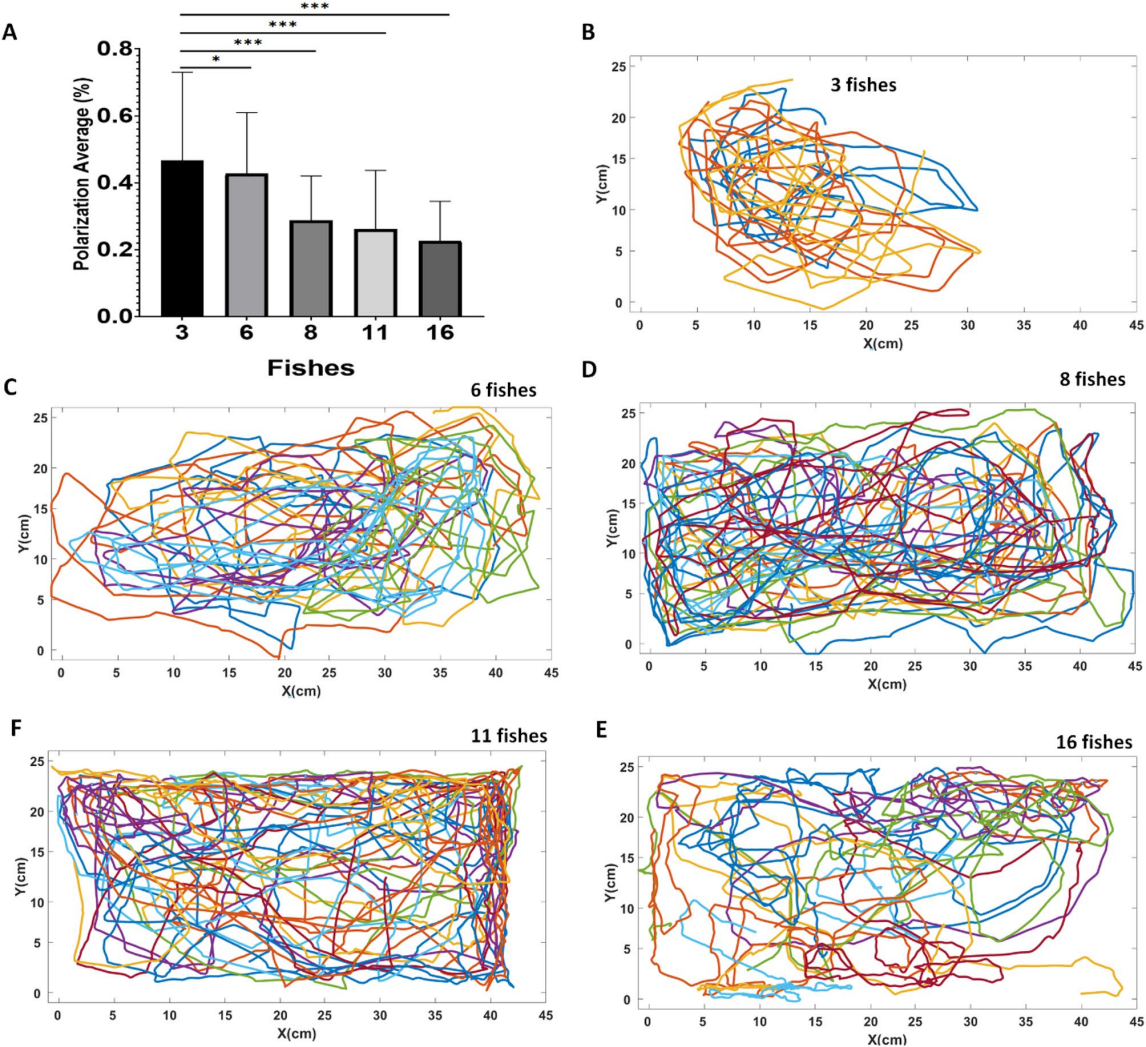
Polarization in groups (N = 3, 6, 8, 11 and 16 fishes) of different sizes of zebrafish. The horizontal lines of each group denote the mean ± SD and the asterisks on the brackets indicate statistical difference in comparison with the frequency of fish between the attempt and the control group by student t test (*p < 0.05; **p < 0.001, ***p < 0.0001).

Then, we tested how the size of the fish group (N = 3, 6, 8 and 11, 16) affects the behavior inside the tank (Fig. 5A). We noticed that the polarization decreases as the groups are increased, during the free swim. Thus, the larger groups showed low and rapidly variable polarization (group with N = 3, mean ± SD: 0.466 ± 0.2643, variation of 56.69%; group with N = 6, mean ± SD: 0.427 ± 0.18 , variation of 42.73%; group with N = 8, mean ± SD: 0.288 ± 0.132, variation of 45.81%; group with N = 11, mean ± SD: 0.262 ± 0.174, variation of 66.64%, group with N = 16, mean ± SD: 0.226 ± 0.11, variation of 52.15%), with significant differences as the group of fish increased in size (${F}_{\mathrm{4,3}}$ = 204.6 , *p* < 0.0001). Figure 5B–F show the zebrafish freestyle traces in different groups, respectively. Note that fish in smaller groups have firmer clusters and more crowded swimming.

### Exploration in the tank

**Figure 6:**
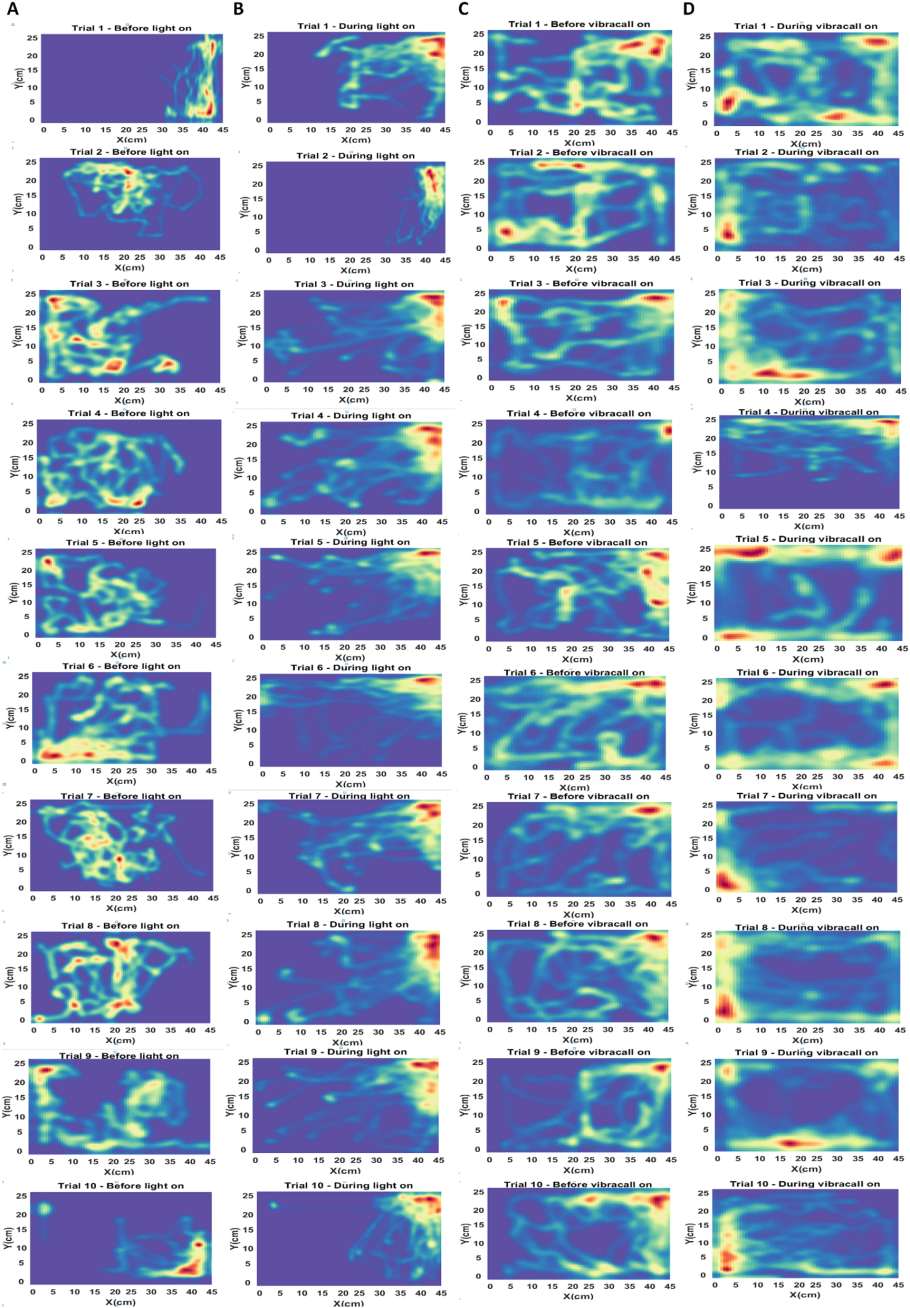
Thermal maps of the zebrafish’s location during complex conditioning (red and vibracall). The heat map contains records of the zebrafish’s location during training. At the moment when the stimulus was triggered, most fish moved to the delimited area of each stimulus, being the right side for the red LED light stimulus and the left side for the vibracall stimulus. (**A**) location of the fish before the red LED light was on, (**B**) location of the fish while the red LED light was on, (**C**) location of the fish before the vibracall stimulus on and D) location of the fish during the vibracall stimulus on.

The heat map developed based on the spatial position of the fish in the periods of complex training (before and during the connected stimuli), showed the fish's intention during the attempts. For comparative purposes, in Fig. 6, there are the heat maps of the red light training (feeder positioned on the right side of the aquarium, Fig. 6B, the vibracall (feeder positioned on the left side of the aquarium, Fig. 6D and the control groups (Fig. 6A,C), corresponding to attempts 1 to 10. It is observed that the fish changed the behavior during the consecutive attempts. Thus, during the period of the red light on Fig. 6B, most fish moved to the right side waiting for food. However, in the period when the vibracall was initially activated, the fish seemed to swim more confused to position themselves on the side corresponding to the stimulus (left side, Fig. 6D, and only in attempt 7 did the fish learn the correct location to wait for food.

### Interaction network in zebrafish

**Figure 7:**
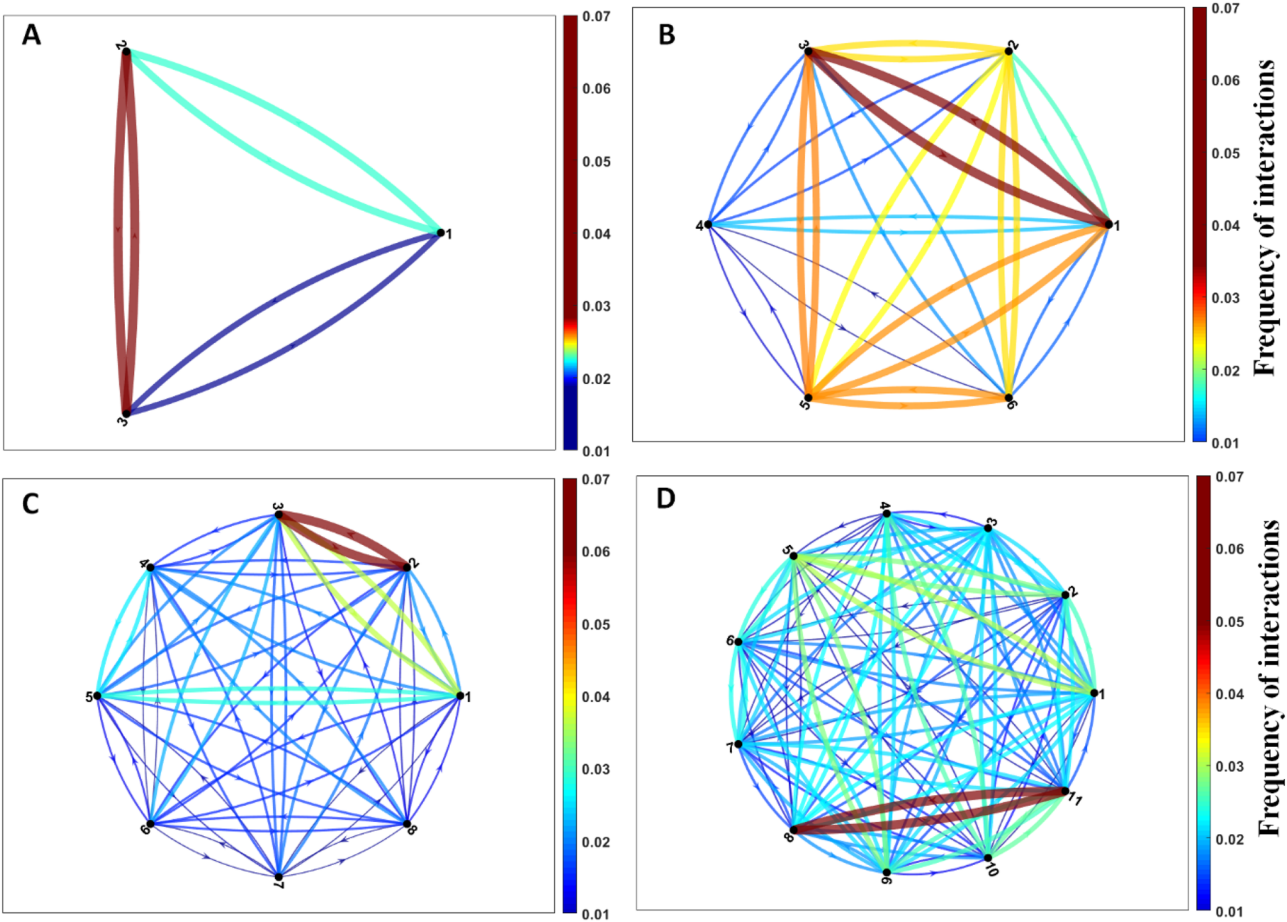
Network of zebrafish shoal interactions. Mutual information was measured from the difference in the angle of the fish's head during movement in the video. Two arrows connect two fish during locomotor activity, being the response of the influence of one fish to another target fish. The line thickness represents the frequency of interactions. Frequency of interactions of the group with three fish (**A**), six fish (**B**), eight fish (**C**) and eleven fish (**D**).

To evaluate the processing of information distributed among the fish in the tank, mutual information was used, the difference in the angle of the fish's head during the movement between the frames. Figure 7 shows the behavioral dynamics of the school, the arrow between the nodes indicates that the fish triggered a locomotor change, and the thickness of this arrow is the value of the measure of mutual information between a pair of fish, measured by the frequency of interactions between fish. Thus, the most active fish influence the behavior change of the least active fish. It is observed that the groups with larger quantities of fish seemed to be more stable in the behavior change (groups with 8 and 11 fish, Fig. 7C,D), while the groups with smaller quantities of fish presented a greater change in behavior (Fig. 7A,B). In addition, at least one fish in each group changes the interaction with the other fish, which can be considered the dominant fish in the school. Evaluating the communication of the group of fish during the conditioned training (group with 16 fish, supplementary file), it was observed that during the conditioning period by the red light, the fish seemed to have more stable interactions in relation to the conditioning period with the vibracall, suggesting that more fish influenced the dynamics of the behavior of others to associate the stimulus with food (see Figure S2).

## Discussion

In this study, a fully automatic behavioral assessment system was developed to measure the zebrafish response in two behavioral perceptions: simple and complex conditioning. The environment automatically delivered stimuli and food during a zebrafish conditioning task.

In addition, the developed algorithm verified the videos captured from the established task, analyzing the fish individually and in groups, presenting an assessment of the behavioral dynamics of the conditioned fish. Although there are some automatic zebrafish conditioning systems^5–7,48–52^, it is important to develop adjustable equipment for the automated conditioning protocol. Usually, several behavioral analysis algorithms are developed independently, such as the available tracking software^4,12,25–31^.

After processing the data, our results demonstrate that the zebrafish learned quickly in simple conditioning, which made an effective association between a conditioned light stimulus and a food reward. In this way, the zebrafish was able to learn from the second experimental attempt at appetizing conditioning (Fig. 2). However, another survey with similar devices showed that the zebrafish learned with at least five daily training attempts^7^. In the most complex assessment, the zebrafish encountered difficulties in some attempts with the vibracall stimulus during the association of the stimulus triggered to the corresponding compartment. However, they were successful in relating the stimulus to a red LED (Fig. 3). Studies show that zebrafish are able to differentiate colors^13,17,19^, and here, that the zebrafish can learn properly from red LED light stimuli.

Previous studies have shown that zebrafish can form double associations (stimulus and environment). In this, the fish can associate both the triggered stimulus time with a food reward and between the reward and its location^7,26,51^. In this way, we divided the aquarium area into four regions, two areas being considered for conditioning (Fig. 3F). This strategy was used to avoid bias in the analysis of the results. Furthermore, it is still not entirely clear whether the zebrafish formed a cognitive map of the environment or just formed associations between a limited space for food (such as the area of the food compartment) and the location of the feeder^7,26^. In this study, in simple conditioning, the fish started to move constantly to the delimited arena, from the second attempt, waiting for more food (Fig. 3). In the most complex training, however, the fish quickly associated to the light stimuli in all attempts, having more difficulties with the vibracall stimulus, as shown in the thermal map (Fig. 6).

It was also observed that the zebrafish group that received the associative stimulus had greater polarity in relation to the control group (Fig. 4A,B). The fish were agitated during the connected stimulus and quickly headed for the food conditioning area, so the group movement coordination was firmer. Although the zebrafish has different polarization distributions for each group of fish formed, these groups can be organized as “shoals”, which are only aggregates of individuals, or “schools”, where the aggregates of individuals have a synchronized and highly polarized movement.

Other characteristics of the movement of the fish group can vary between shoals and schools, with the zebrafish school being faster and less dense compared to the zebrafish shoals^52^. In that study, it was observed that polarization was stronger in stimulated groups than in control groups. In addition, the groups with smaller amounts of fish showed greater polarization, the fish were swimming in the same difference and clusters (Fig. 5). The increase in the group may influence the dynamics of the fish school. Thus, in other models of collective movement, the polarization of a group takes into account its size or density^52,53^.

When analyzing the dynamics of the interaction network between the fish during the conditioning, it was noticed that the vibracall stimulus left the fish more agitated and consequently exchanged more information, influencing the dynamics of the movements of the fish school (Fig. 7 and Supplementary Information S2). Thus, it was noticed that the most active fish influence the change in the behavior of less active fish. And that larger groups of fish seemed to be more stable in changing their behavior.

Automated zebrafish monitoring offers advantages for more accurate behavioral analysis compared to manual systems, ensuring more controlled work and without human intervention (See Video 1, Supplementary File). The proposed system can clearly assess the interest and behavioral dynamics of fish individually and organized in groups. Thus, our system allows for the monitoring of fish in conditioned and free movements, reducing human invasiveness in research. In addition, it is a fully automatic system that can be adjusted according to a chosen protocol. Here, we chose conditioning by two stimuli, but it is easily adaptable to other approaches, or approaches that take into account video processing and behavior analysis, being established in real time or after training. Some automatic control tools may be limited to experiments and not adjustable for other experiments. In this system, we can adjust for various experiences such as assessing behavior for toxicity, sleep, learning by aversion and several other behaviors that require video processing. In addition, this tool can be used for other species of smaller fish, however it will be necessary to train YOLOv2 with data from new fish.

## Conclusion

A new automated system for real-time assessment of zebrafish conditioning was presented here. It was noticed that the zebrafish are able to learn with just two consecutive attempts. There was a difference in learning in relation to the red led light and vibracall stimuli, where fish learned more easily when they were stimulated by red LED light. Conditioned fish showed high polarization in relation to unstimulated fish. In addition, the higher polarization is related to smaller groups of fish, showing that the group dynamics are more stable and coordinated. It is believed that the methodology used will serve for future evaluations of behavioral applications of zebrafish research, not only for conditioning fish, but for other behavioral analyzes such as toxicity, sleep, aversion learning and several other behaviors that demand the processing of video, adjusting the evaluation protocol. In addition, this tool can be used for other species of smaller fish, however it will be necessary to train YOLOv2 with data from new fish.

## Supplementary Information

Supplementary Information.

## Data Availability

Source data are provided with this paper (Supplementary information).

## References

[1] Pérez-Escudero A, Vicente-Page J, Hinz RC, Arganda S, de Polavieja GG (2014). idTracker: tracking individuals in a group by automatic identification of unmarked animals. Nat. Methods.

[2] Guttridge TL, Myrberg AA, Porcher IF, Sims DW, Krause J (2009). The role of learning in shark behaviour. Fish Fish..

[3] Magurran AE, Higham A (1988). Information transfer across fish shoals under predator threat. Ethology.

[4] Chacon DM, Luchiari AC (2014). A dose for the wiser is enough: the alcohol benefits for associative learning in zebrafish. Prog. Neuro-Psychopharmacol. Biol. Psychiatry.

[5] Parker MO (2013). Development and automation of a test of impulse control in zebrafish. Front. Syst. Neurosci..

[6] Parker MO, Millington ME, Combe FJ, Brennan CH (2012). Development and implementation of a three-choice serial reaction time task for zebrafish (*Danio rerio*). Behav. Brain Res..

[7] Doyle JM (2017). A simple automated system for appetitive conditioning of zebrafish in their home tanks. Behav. Brain Res..

[8] Bilotta J, Risner ML, Davis EC, Haggbloom SJ (2005). Assessing appetitive choice discrimination learning in zebrafish. Zebrafish.

[9] Manabe K, Dooling RJ, Takaku S (2013). An automated device for appetitive conditioning in zebrafish (*Danio rerio*). Zebrafish.

[10] Engeszer RE, Patterson LB, Rao AA, Parichy DM (2007). Zebrafish in the wild: a review of natural history and new notes from the field. Zebrafish.

[11] Parker MO (2014). The utility of zebrafish to study the mechanisms by which ethanol affects social behavior and anxiety during early brain development. Prog. Neuro-Psychopharmacol. Biol. Psychiatry.

[12] Sison M, Gerlai R (2010). Associative learning in zebrafish (*Danio rerio*) in the plus maze. Behav. Brain Res..

[13] Avdesh A (2012). Evaluation of color preference in zebrafish for learning and memory. J. Alzheimer’s Dis..

[14] Fero K, Yokogawa T, Burgess HA (2011). The behavioral repertoire of larval zebrafish. NeuroMethods.

[15] Tegelenbosch RAJ, Noldus LPJJ, Richardson MK, Ahmad F (2012). Zebrafish embryos and larvae in behavioural assays. Behaviour.

[16] De Marco RJ, Groneberg AH, Yeh CM, Treviño M, Ryu S (2014). The behavior of larval zebrafish reveals stressor-mediated anorexia during early vertebrate development. Front. Behav. Neurosci..

[17] Spence R, Smith C (2008). Innate and learned colour preference in the zebrafish, *Danio rerio*. Ethology.

[18] Mueller KP, Neuhauss SCF (2012). Automated visual choice discrimination learning in zebrafish (*Danio rerio*). J. Integr. Neurosci..

[19] Roy T (2019). Color preferences affect learning in zebrafish, *Danio rerio*. Sci. Rep..

[20] Luchiari AC, Chacon DMM (2013). Physical exercise improves learning in zebrafish, *Danio rerio*. Behav. Process..

[21] Zeddies DG, Fay RR (2005). Development of the acoustically evoked behavioral response in zebrafish to pure tones. J. Exp. Biol..

[22] Bhandiwad AA, Zeddies DG, Raible DW, Rubel EW, Sisneros JA (2013). Auditory sensitivity of larval zebrafish (*Danio rerio*) measured using a behavioral prepulse inhibition assay. J. Exp. Biol..

[23] Cervi AL, Poling KR, Higgs DM (2012). Behavioral measure of frequency detection and discrimination in the zebrafish, *Danio rerio*. Zebrafish.

[24] Neo YY (2015). Behavioral changes in response to sound exposure and no spatial avoidance of noisy conditions in captive zebrafish. Front. Behav. Neurosci..

[25] Al-Imari L, Gerlai R (2008). Sight of conspecifics as reward in associative learning in zebrafish (*Danio rerio*). Behav. Brain Res..

[26] Karnik I, Gerlai R (2012). Can zebrafish learn spatial tasks? An empirical analysis of place and single CS–US associative learning. Behav. Brain Res..

[27] Sison M, Gerlai R (2011). Associative learning performance is impaired in zebrafish (*Danio rerio*) by the NMDA-R antagonist MK-801. Neurobiol. Learn. Mem..

[28] Bai Y-X (2018). Automatic multiple zebrafish tracking based on improved HOG features. Sci. Rep..

[29] Xu Z, Cheng XE (2017). Zebrafish tracking using convolutional neural networks. Sci. Rep..

[30] Qian Z-M, Cheng XE, Chen YQ (2014). Automatically detect and track multiple fish swimming in shallow water with frequent occlusion. PLoS ONE.

[31] Wang SH, Cheng XE, Qian Z-M, Liu Y, Chen YQ (2016). Automated planar tracking the waving bodies of multiple zebrafish swimming in shallow water. PLoS ONE.

[32] Thomson JS, Al-Temeemy AA, Isted H, Spencer JW, Sneddon LU (2019). Assessment of behaviour in groups of zebrafish (*Danio rerio*) using an intelligent software monitoring tool, the chromatic fish analyser. J. Neurosci. Methods.

[33] Deakin AG (2019). Automated monitoring of behaviour in zebrafish after invasive procedures. Sci. Rep..

[34] Bossé GD, Peterson RT (2017). Development of an opioid self-administration assay to study drug seeking in zebrafish. Behav. Brain Res..

[35] Baker MR, Wong RY (2019). Contextual fear learning and memory differ between stress coping styles in zebrafish. Sci. Rep..

[36] Vignet C (2013). Systematic screening of behavioral responses in two zebrafish strains. Zebrafish.

[37] Zarantoniello M (2020). Zebrafish (*Danio rerio*) physiological and behavioural responses to insect-based diets: a multidisciplinary approach. Sci. Rep..

[38] de Almeida Moura C, da Silva Lima JP, Silveira VAM, Miguel MAL, Luchiari AC (2017). Time place learning and activity profile under constant light and constant dark in zebrafish (*Danio rerio*). Behav. Process..

[39] Percie du Sert N (2020). The ARRIVE guidelines 2.0: updated guidelines for reporting animal research. PLOS Biol..

[40] You M-S (2020). Red LED light treatment promotes cognitive learning through up-regulation of trpm4 in zebrafish. J. Photochem. Photobiol. B Biol..

[41] Redmon, J. & Farhadi, A. YOLO9000: better, faster, stronger. In *2017 IEEE Conference on Computer Vision and Pattern Recognition (CVPR)* 2017-Janua, 6517–6525 (IEEE, 2017).

[42] Liu Z, Chen Z, Li Z, Hu W (2018). An efficient pedestrian detection method based on YOLOv2. Math. Probl. Eng..

[43] de Oliveira Barreiros M, de Oliveira Dantas D, de Oliveira Silva LC, Ribeiro S, Barros AK (2021). Zebrafish tracking using YOLOv2 and Kalman filter. Sci. Rep..

[44] Romero-Ferrero F, Bergomi MG, Hinz RC, Heras FJH, de Polavieja GG (2019). Idtracker.ai: tracking all individuals in small or large collectives of unmarked animals. Nat. Methods.

[45] Butail S, Ladu F, Spinello D, Porfiri M (2014). information flow in animal–robot interactions. Entropy.

[46] Niizato T (2020). Finding continuity and discontinuity in fish schools via integrated information theory. PLoS ONE.

[47] Crosato E (2018). Informative and misinformative interactions in a school of fish. Swarm Intell..

[48] de Chazal P, Penzel T, Heneghan C (2004). Automated detection of obstructive sleep apnoea at different time scales using the electrocardiogram. Physiol. Meas..

[49] Manabe K, Dooling RJ, Takaku S (2013). Differential reinforcement of an approach response in zebrafish (*Danio rerio*). Behav. Process..

[50] Cerutti DT, Jozefowiez J, Staddon JER (2013). Rapid, accurate time estimation in zebrafish (*Danio rerio*). Behav. Process..

[51] Braubach OR, Wood H-D, Gadbois S, Fine A, Croll RP (2009). Olfactory conditioning in the zebrafish (*Danio rerio*). Behav. Brain Res..

[52] Miller N, Gerlai R (2012). From schooling to shoaling: patterns of collective motion in zebrafish (*Danio rerio*). PLoS ONE.

[53] Becco C, Vandewalle N, Delcourt J, Poncin P (2006). Experimental evidences of a structural and dynamical transition in fish school. Phys. A Stat. Mech. Appl..

