# Supplementary material for: Zebrafish automatic monitoring system for conditioning and behavioral analysis
Source: Sci Rep. 2021 Apr 29;11:9330. doi: 10.1038/s41598-021-87502-6 (PMC8085222; doi:10.1038/s41598-021-87502-6)
Supplement: Supplementary file 1 — Supplementary Information. [file 41598_2021_87502_MOESM1_ESM.docx]

**SUPPLEMENTARY INFORMATION**

**Zebrafish Automatic Monitoring System for Conditioning and Behavioral Analysis**

¹Marta de Oliveira Barreiros, ¹Felipe Gomes Barbosa, ¹Diego de Oliveira Dantas, ¹Daniel Luna dos Santos, ²Sidarta Ribeiro, ³Giselle Cutrim Santos, ¹Allan Kardec Barros

[marta-barreiros@hotmail.com](mailto:marta-barreiros@hotmail.com); [felipegomes.mj@gmail.com](mailto:felipegomes.mj@gmail.com), [diego_odantas@hotmail.com](mailto:diego_odantas@hotmail.com); [dantmec@gmail.com](mailto:dantmec@gmail.com); sidartaribeiro@neuro.ufrn.br; [giselle.cutrim@gmail.com](mailto:giselle.cutrim@gmail.com); [akduailibe@gmail.com](mailto:akduailibe@gmail.com)

¹Department of Electrical Engineering, Laboratory for Biological Information Processing (PIB), Federal University of Maranhão (UFMA), São Luís-MA, Brazil.

² Brain Institute, Federal University of Rio Grande do Norte, Natal-RN, Brazil

³ Department of Biology Sciences, State University of Maranhão, São Luís-MA, Brazil.

Corresponding author: M.B.

Department of Electrical Engineering, Federal University of Maranhão (UFMA)

Av. dos Portugueses, 1966, Vila Bacanga,

CEP 65080-805

São Luís - MA, Brazil

Phone/Fax: +55 98 3272 8220;

E-mail: **marta-barreiros@hotmail.com**

S1 - Stage of the zebrafish conditioning system. The zebrafish behavioral evaluation algorithm consists of the following stages: fish conditioning process, video processing, fish detection and behavioral tracking and modeling. From the tracking it is possible to measure the average polarization, speed, distance traveled, network of interaction between the fish and to create the fish route by heat map.


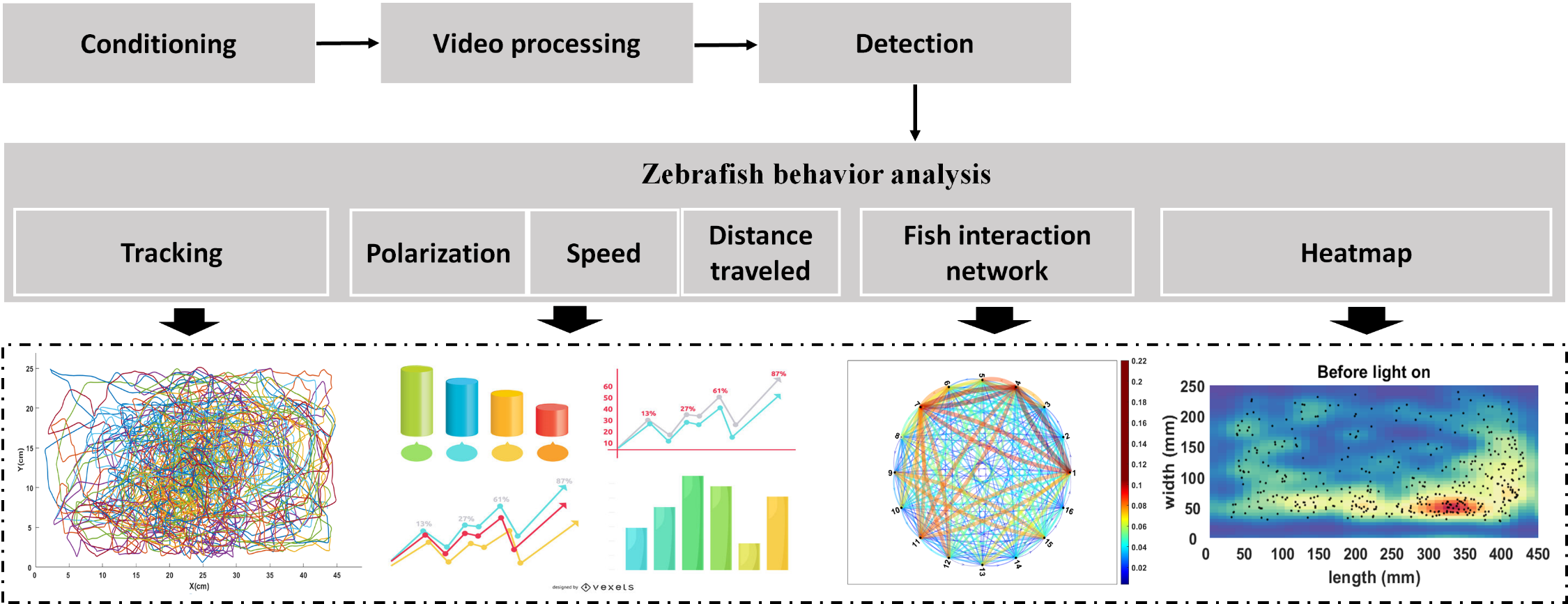


S2 - Zebrafish school interaction network. The interaction network of the fish school was created by mutual information of the difference in the angle of the fish's head during the movement between the frames. The arrow between the nodes indicates that the fish has triggered a locomotor change, and the thickness of this arrow is the value of the measure of mutual information between a pair of fish, measured by the frequency of interactions between the fish. Thus, the behavioral dynamics of the school shows that during the vibracall stimulus, the fish showed greater interaction of movement. A) Interaction between the fish during the conditioning period with a red LED light stimulus. B) Interaction between fish during the conditioning period with vibracall stimulus.


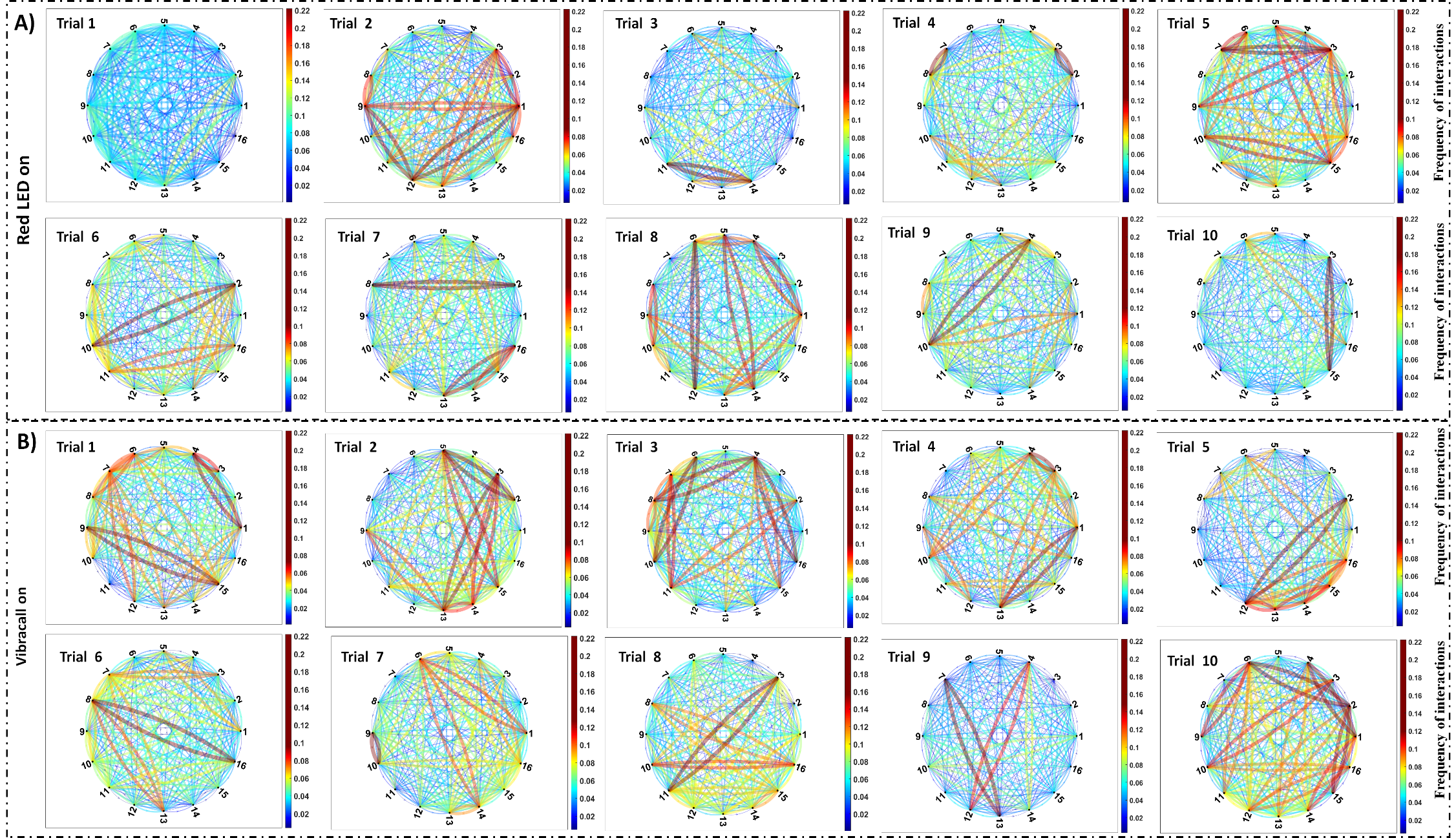


**SUPPLEMENTARY VIDEO**

V1 - Video of the online training system. The video shows a simulation of food control and video processing in real time, including detection and tracking.

<https://drive.google.com/file/d/1PcyXhpnjGDrh__ObACRMkEyu7hEu8XUM/view?usp=sharing>

V2-V11 - Food conditioning with red LED light stimulus in attempts 1 to 10.

<https://drive.google.com/drive/folders/1rqBlZvGFuLU0mSdxGs9o2Tz63vVylVcf?usp=sharing>

V12– V21 - Control group during red LED light stimulus in trial 1 to 10.

<https://drive.google.com/drive/folders/1z5cp0ORenznDtAqNYy_3OJl7ldyO7Odq?usp=sharing>

V22– 31 - Food conditioning with vibracall stimulus in trial 1 to 10.

<https://drive.google.com/drive/folders/19p8AqgMk8CIOXpzmBP0ai9ZsA6YdyWgW?usp=sharing>

V32– 41 - Control group during vibracall stimulus in trial 1 to 10.

<https://drive.google.com/drive/folders/1C9plUjBVd30cjRDln1VrDp1FnbecmRr6?usp=sharing>

All vídeos:

https://drive.google.com/drive/folders/1wBk4MkS03TWKUML4Gmy8WWktjf5-KgG5
